# Supplementary material for: Polymorphisms in Pfkelch13 domains before and after the introduction of artemisinin-based combination therapy in Southwest Nigeria
Source: PLoS One. 2025 Mar 31;20(3):e0316479. doi: 10.1371/journal.pone.0316479 (PMC11957316; doi:10.1371/journal.pone.0316479)
Supplement: Supporting information 2 — (ZIP) [file pone.0316479.s002.zip › 050KN2F_PREMIX_Plate_KELCH2_A05.pdf]

Samples: 13375
Bases: 1133
Average spacing: 12

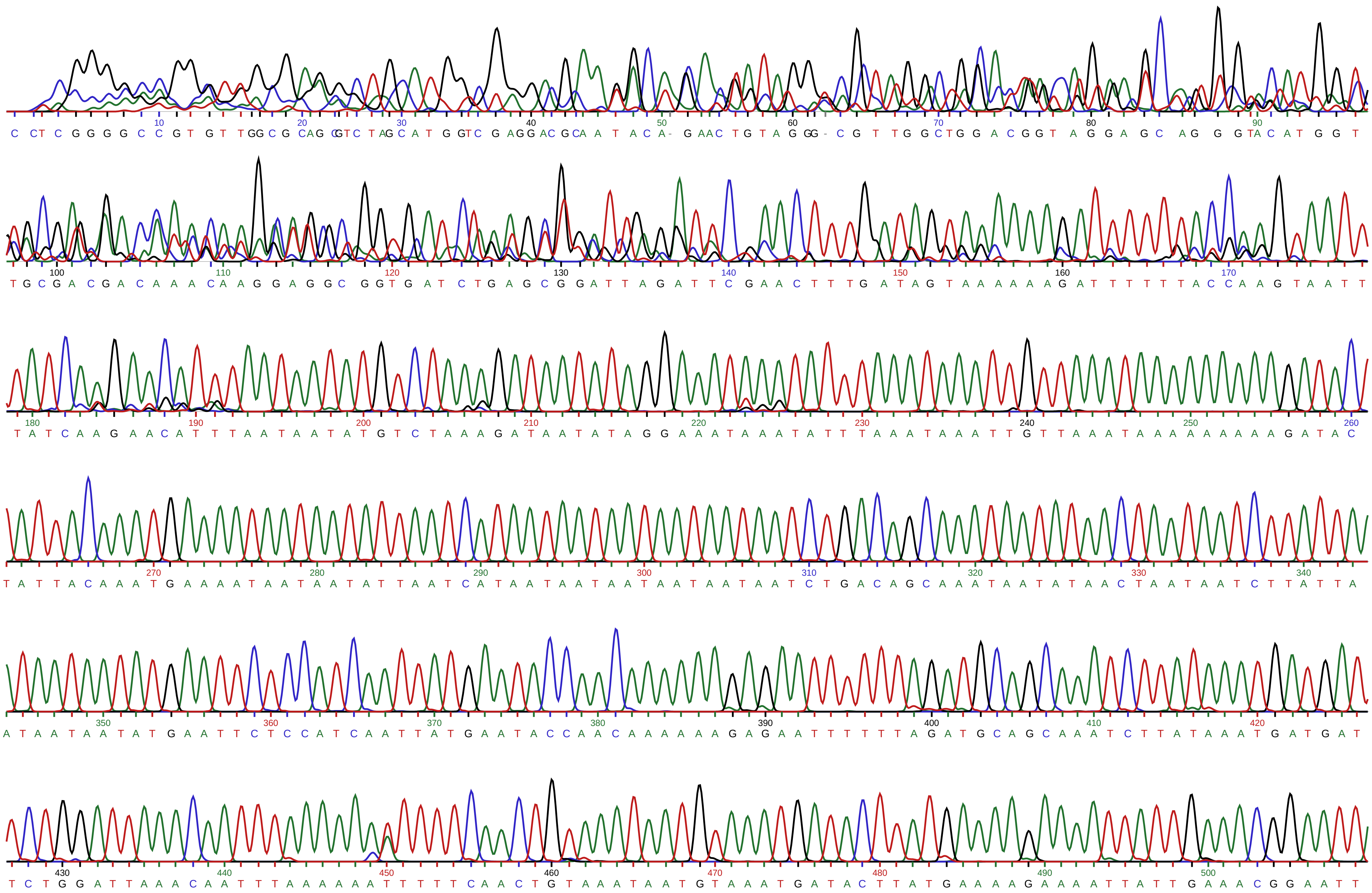

Samples: 13375
Bases: 1133
Average spacing: 12

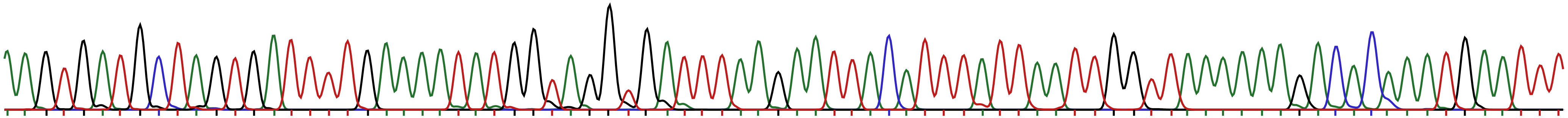

510 520 530 540 550 560 570 580 590

A A G T G A T G C T A G T G A T T T T G A A A A T A T G G T A G G T G A T T T A A G A A T T A C A T T T A T T A A T T G G T T A A A A A A G A C A C A A A T G A A T T T

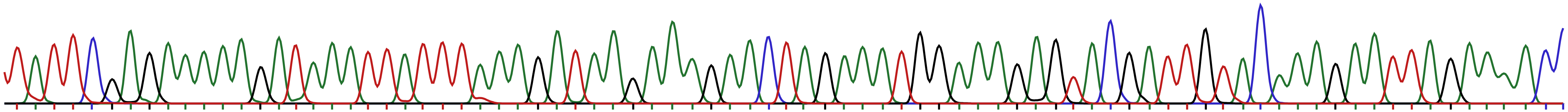

600 610 620 630 640 650 660 670

T A T T C G A G A A A A A G A T A A A T T A T T T A A A G A T A A G A A A G A A C T A G A A A T G G A A A G A G T A C G A T T G T A C A A A G A A T T A G A A A A C C

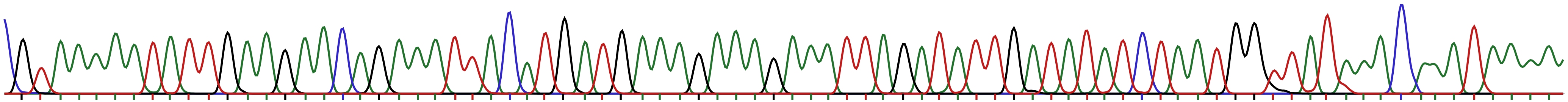

680 690 700 710 720 730 740 750

G T A A A A A T A T T G A A G A A C A G A A A T T A C A T G A T G A A A G A A A G A A A T T A G A T A T T G A T A T A T C T A A T G G T T A T A A A C A A A T A A A A

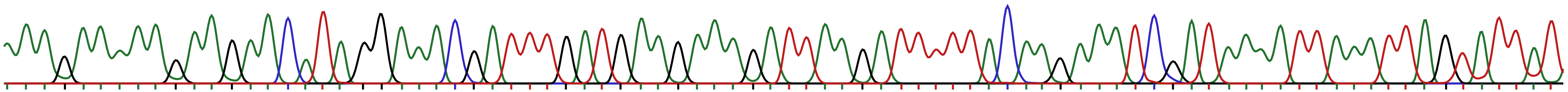

760 770 780 790 800 810 820 830 840

A A A G A A A A A G A A G A A C A T A G G A A A C G A T T T G A T G A A G A A A G A T T A A G A T T T T T A C A A G A A A T C G A T A A A A T T A A A T T A G T A T T A T

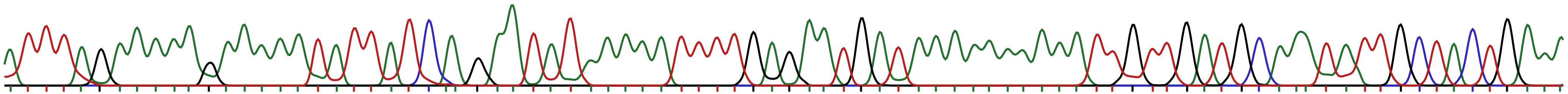

850 860 870 880 890 900 910 920 930

A T T T A G A A A A A G A A A A A T A T T A T C AA G A A T A T A A A A A T T T T G A G A A T G A T A A A A A A A A A A T T G T T G A T G C A AA T A T T G C T A C T G A A A

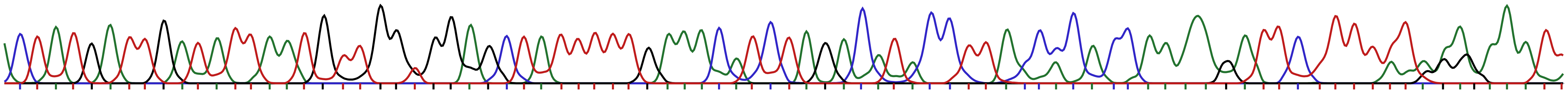

940 950 960 970 980 990 1000 1010

C T A T G A T T G A T A T T A A T G T T G G T G G A G C T A T T T T T G A A A C A T C T A G A C A T A C C T T A C C A C A C C A A A A G A T T C T T T T T T A G A G A A A T T

Samples: 13375
Bases: 1133
Average spacing: 12

8/17/2022

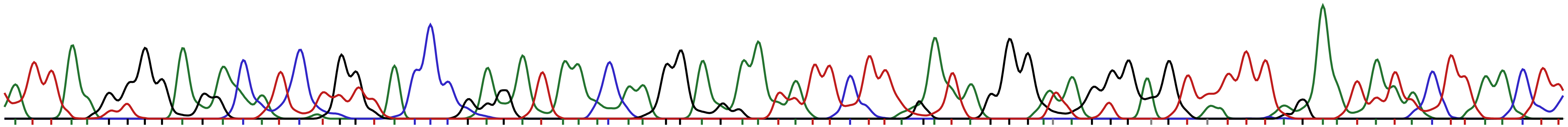

1020 1030 1040 1050 1060 1070 1080 1090 1100

A T T A A G G G G A G A C A T C T G G T A C C C G A G A T A A A C A A G G A G A A T A T T C T T A G A T A G G G A - A G G G - G T - T T T A G A A T A T A C T T A A C T T

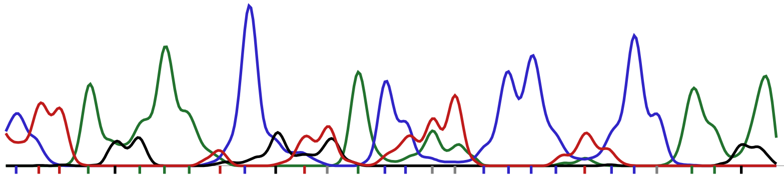

1110 1120 1130

C T T A G A A A T C G T - A C C - - C C C C T C C - A A G
